# lncRNA CASC7 regulates pathological progression of ox-LDL-stimulated atherosclerotic cell models via sponging miR-21 and regulating PI3K/Akt and TLR4/NF-κB signaling pathways

**DOI:** 10.18632/aging.203757

**Published:** 2021-12-09

**Authors:** Xueliang Pei, Yongjin Wen, Facai Cui, Zhiyuan Yang, Zhouliang Xie

**Affiliations:** 1 Department of Cardiovascular Surgery, Fuwai Central China Cardiovascular Hospital, Henan Provincial People’s Hospital, Zhengzhou, Henan, China; 2 Department of Clinical Laboratory, Henan Provincial People’s Hospital, People’s Hospital of Zhengzhou University, Zhengzhou, Henan, China

**Keywords:** atherosclerosis, CASC7, miR-21, PI3K/Akt, TLR4/NF-κB, proliferation

## Abstract

Atherosclerosis (AS) is a frequently occurring cause of cardiovascular disease and involves a complicated pathophysiological process. Studies suggest that long non-coding RNAs (lncRNAs) are involved in AS genesis and progression, but mechanisms underlying these connections are unclear. Therefore, this work focused on exploring the role of lncRNA CASC7 in AS. In this study, RNA-seq sequencing results identified 1040 lncRNAs differentially expressed between AS patients and healthy controls. Of these lncRNAs, 458 were up-regulated and 582 were downregulated. CASC7 was found to be down-regulated in serum samples from AS patients and in HUVEC and VSMC exposed to ox-LDL. Overexpression of CASC7 inhibited proliferation and enhanced apoptosis of VSMC, and it markedly reduced IL-1β, IL-6 and TNF-α levels in HUVEC. Increased expression of a CASC7 target, miR-21, abolished the effects of CASC7 on HUVEC and VSMC. Notably, miR-21 targets PI3K in VSMC and TLR4 in HUVEC. The inhibitory effect of CASC7 was decreased by stimulation of PI3K, suggesting that the CASC7/miR-21 axis functions through PI3K/Akt signaling in VSMC. Similarly, the inhibitory effect of CASC7 on the inflammatory response in HUVEC was abolished through activating the TLR4/NF-κB signaling pathway. CASC7 inhibited proliferation and enhanced the apoptosis of VSMC through modulating the miR-21/PI3K-AKT axis, and upregulating CASC7 suppressed the inflammatory response of HUVEC by sponging miR-21 to inhibit the TLR4/NF-κB signal pathway.

## INTRODUCTION

Atherosclerosis (AS) is a form of cardiovascular disease in which disorders of lipid and cholesterol metabolism lead to chronic arterial wall inflammation. AS is a leading global cause of morbidity and mortality [1, 2]. Phenotypic differentiation and abnormal growth of vascular smooth muscle cells (VSMC) are associated with early development of AS and damage to blood vessels [3]. In addition, dysfunctions in endothelial cells contribute to the chronic inflammatory response and alterations of the redox balance inside the arterial wall, and these results are considered to be crucial factors in the pathogenesis of AS [4, 5]. The aberrant metabolism and resulting accumulation of oxidized low-density lipoprotein (ox-LDL) is another noted risk factor for AS [6, 7]. Moreover, ox-LDL modulates VSMC growth and apoptosis as well as endothelial inflammation during the AS pathological process [8–10]. Therefore, exploring the underlying mechanisms in which ox-LDL regulates functions of VSMC and endothelial cells would help to reveal ways that ox-LDL induces AS.

Members of a class of RNAs called long noncoding RNAs (lncRNA) have been found to contribute to AS. These RNAs are more than 200 nucleotides in length and do not encode protein. Specific lncRNAs have been shown to be involved in the biological processes leading to AS by regulating the inflammatory response, cell proliferation, apoptosis and migration [11–13]. For example, a recent study reported that expression of the lncRNA maternally expressed gene 3 (MEG3) decreases within VSMC exposed to ox-LDL, while suppressing its expression enhances the proliferation of ox-LDL-exposed VSMC and reduces apoptosis in the context of AS [14]. Another lncRNA, small nucleolar RNA host gene 6 (SNHG6) likely acts as a promoter of AS, as it has been shown to reduce human umbilical vein endothelial cell (HUVEC) apoptosis, oxidative stress and inflammation resulting from ox-LDL treatment [15]. The lncRNA cancer susceptibility candidate 7 (CASC7), with a molecular weight of 9.3 kb, can modulate cell migration and growth in certain cancer types [16, 17]. Recent studies have reported that CASC7 suppresses myocardial apoptosis in the context of myocardial ischemia-reperfusion and represses the secretion of inflammatory cytokines [18, 19]. However, potential effects of CASC7 on AS have not yet been addressed.

A growing number of studies indicate that the competing endogenous RNA (ceRNA) network, which involves the inhibition of the functions of microRNA (miRNA) and mRNA, accounts for a vital pathway that helps to explain the functions of lncRNAs. In particular, MEG3 can regulate the balance between VSMC proliferation and apoptosis in AS through modulating the miR-26a/Smad1 Axis [20]. Similarly, an lncRNA known as myocardial infarction associated transcript (MIAT) suppresses efferocytosis in late AS through increasing CD47 expression via sponging miR-149-5p. Recently, miR-21 has been reported to mediate many pathophysiological mechanisms of CASC7, such as the enhancing of corticosteroid sensitivity [18], the inhibition of myocardial apoptosis and the suppression of colon cancer cell growth and invasion [21]. Interestingly, miR-21 may accelerate the development of AS by enhancing plaque necrosis, cell apoptosis, as well as vascular inflammation in the process of AS [22, 23]. Despite these intriguing connections, associations of the CASC7/miR-21 axis with the progression of AS remain to be clarified.

The pathway involving phosphatidyl inositide 3-kinase (PI3K) and the serine/threonine protein kinase AKT and the pathway involving toll-like receptor 4 (TLR4) and nuclear factor kappa B (NF-κB) are central targets of miR-21. For example, Lu et al. discovered that miR-21 suppressed the proliferation and new blood formation of vascular endothelial cells by regulating the PI3K/Akt pathway [24], and Zhao et al. identified TLR4/NF-κB signaling as a target of miR-21 in alleviating high glucose-induced inflammation [25]. Meanwhile, the PI3K/Akt and TLR4/NF-κB signaling pathways also have been reported to be involved in increased lipid deposition, decreased collagen content and up-regulation of the release of inflammatory factors, such as matrix metalloproteinase 9, interleukin-6 (IL-6), C-reactive protein and tumor necrosis factor-α (TNF-α), in the pathological progression of AS [26]. But whether PI3K/Akt and TLR4/NF-κB pathways account for mechanisms associated with CASC7/miR-21 axis is unknown.

Ox-LDL has been reported in several *in vitro* studies to be a stimulating factor that induces apoptosis [27, 28]. Therefore, ox-LDL was used to stimulate HUVEC and VSMC in this work to establish an *in vitro* model of AS. This study also detected CASC7 levels and analyzed the roles of CASC7 in physiological functions of VSMC and HUVEC, including proliferation, apoptosis and inflammatory responses. Finally, the underlying function of the TLR4/NF-κB and PI3K/Akt pathways within the CASC7/miR-21 axis was analyzed.

## MATERIALS AND METHODS

### Patients

Serum samples were collected from 30 healthy volunteers and 30 AS patients at Henan Provincial People’s Hospital (Zhengzhou, China) from May 2017 to March 2020. Each of the collected samples was frozen at once in liquid nitrogen and was stored at −80°C prior to subsequent analysis. Serum samples were collected from 5 AS patients and 5 healthy volunteers, and total RNA was extracted. The cDNA was synthesized using random primers, reverse transcriptase, DNA polymerase I and RNase H. The IlluminaHiSeq library fragments were purified by AMPre XP system (Beckman Coulter, Beverly, MA) after adenosylated DNA fragments were hybridized. The IlluminaHiSeq library fragments were sequenced by double-end sequencing (Beijing Boao Biotechnology Co., Ltd, Beijing, China). The Ethics Committee of Henan Provincial People’s Hospital approved the study protocols.

### Cell culture, ox-LDL treatment and transfection

Human VSMC and HUVEC were provided by the Chinese Academy of Sciences (Beijing, China). HUVEC and VSMC were cultivated in Dulbecco’s modified Eagle’s medium (DMEM) (Sigma, St. Louis, MO, USA) supplemented with 10% fetal bovine serum and 0.1% streptomycin and were incubated in a humid 5% CO_2_ environment at 37°C.

Ox-LDL-treated HUVEC and VSMC were used to construct an AS cell model. Untransfected VSMC were exposed to ox-LDL (Beyotime, Beijing, China) of various concentrations (0, 25, 50, 100, or 200 mg/L) for a period of 24 h. After transfection, cells were treated with 100 mg/L ox-LDL for a period of 24 h to study the effects of CASC7 on the physiological activity of VSMC and HUVEC.

For lncRNA CASC7 overexpression, a vector directing the expression of lncRNA CASC7 and a corresponding negative control (NC) RNA were obtained from GenePharma (Shanghai, China). CASC7 and NC were transfected into VSMC and HUVEC with Lipofectamine RNAiMAX (Invitrogen, Carlsbad, CA, USA) according to the manufacturer’s instructions.

To overexpress or downregulate miR-21, miR-21 inhibitors and mimics were provided by GenePharma (Shanghai, China). Lipofectamine 2000 (Life Technologies, Carlsbad, CA, USA) was employed for transfection of these constructs, according to the manufacturer’s instructions. The PI3K agonist 740Y-P (25 μmol/L) and TLR4 agonist HY-P1439 (20 μmol/L) were used to analyze the roles of PI3K and TLR4, respectively.

### Cell counting kit-8 (CCK-8) assay

CCK-8 assays were performed to evaluate the growth of HUVEC and VSMC. At 48 h post-transfection, cells were inoculated at a density of 1 × 10^3^ cells/well in 96-well plates and were cultivated in DMEM for a period of 4 h. Then, 100 μL DMEM was mixed with 10 μL CCK8 reagent (Dojindo, Kumamoto, Japan). At 0, 12, 24, and 48 h, proliferation was detected by measuring absorbance at 450 nm with a microplate reader (Berthold Technologies GmbH & Co., KG, Germany).

### Transwell assay

Migration or invasion of VSMC and HUVEC was assessed in Transwell chambers. Briefly, VSMC and HUVEC were inoculated in serum-free media at densities of 5 × 10^6^ cells/well in the upper chamber of the Transwell apparatus. Following 24-hour serum starvation, a cotton swab was used to remove cells remaining in the upper chamber, and a 30 min treatment with 4% paraformaldehyde was used to fix cells on the surface of the lower chamber. Then, fixed cells were stained with 0.1% crystal violet. Finally, migrated VSMC and HUVEC were counted under a microscope at 200× magnification by randomly selecting 5 fields.

### Flow cytometry

The rates of apoptosis of VSMC and HUVEC were tested by flow cytometry. Briefly, when cell confluence in each experimental group reached approximately 80%, the cell culture medium was collected and then cells were digested with trypsin. The cells were gently removed and transferred to a 1.5 mL centrifuge tube, centrifuged at 1000 g for 5 min. Resuspended cells (5 × 10^6^) were centrifuged for 5 min, the supernatant was discarded and cells were resuspended with binding buffer (195 μL). The cell suspension was mixed with staining solution containing annexin V-FITC (5 μL) and propidium iodide (PI, 10 μL). Cells were incubated for 10 to 20 min at ambient temperature (20 to 25°C) in the dark and then analyzed with a flow cytometer.

### Enzyme-linked immunosorbent assay (ELISA)

The levels of inflammatory cytokines, including as TNF-α, IL-6 and interleukin-1β (IL-1β), were measured through ELISA. Briefly, after cells were cultured continuously for 24 h, supernatants were harvested, and cytokines were detected with an ELISA kit (R&D Systems, Abingdon, UK) according to the manufacturer’s instructions. Absorbances at 450 nm were measured with a microplate reader (Rayto Life and Analytical Science).

### RNA extraction and reverse transcription-polymerase chain reaction (qRT-PCR)

Cells were harvested 24 h after transfection. Cells were lysed with Trizol, and RNA was extracted with chloroform and precipitated overnight with isopropanol. For each step, 100 ng total RNA was used. Small RNA was isolated with a miRNA Isolation Kit, and a miScript Reverse Transcription Kit was utilized to synthesize cDNA. The ABI 7500 FAST fluorescent qRT-PCR system was utilized for qRT-PCR analysis. The CASC7 and miR-21 levels in each group were measured. The PCR conditions were 95°C for 5 s and 60°C for 34 s, with a total of 40 cycles. U6 RNA served as an internal control, and the ΔΔCT method was used to determine relative gene expression. Primer sequences utilized in this work are shown in Table 1.

**Table 1:** Sequences of primers for RT-PCR.

**Genes**	**Sequences**	
	**Forward**	**Reverse**
CASC7	5′-ATCAACGTCAAGCTGGGAGG-3′	5′-CTTGTCCCCCGCTCGTTC-3′
POTED	5′-GCAGAATGCCATGGTTTCCC-3′	5′-GGACAGCTGGCAAGAGACTT-3′
CWH43	5′-CGGCGGCAAAGTGCTTACAG-3′	5′-GTGCAGGGTCCGAGGT-3′
DEFB115	5′-CCAAGGCCAACCGCGAGAAGAT-3′	5′-AGGGTACATGGTGGTGCCGCCA-3′
AC073416.2	5′-UCAGUGCAUCACAGAACUUUGU-3′	5′-CACAAATTCCCATCATTCCC-3′
BPY2C	5′-ACACTCCAGCTGGGT CAGTGCATC-3′	5′-CTCAACTGGTGTCGTGGA-3′
miR-21	5′- GTCGTATCCAGTGCAGGGTAACA-3′	5′-CGCGCTAGCTTATCAGACTGA-3′
U6	5′-GCTTCGGCAGCACATATACTAAAAT-3′	5′-CGCTTCACGAATTTGCGTGTCAT-3′

### Luciferase assay

Mutant (MUT) and wild type (WT) 3′-untranslated regions (3′-UTR) of CASC7 that contained predicted miR-21 binding sites were subcloned into to the pmirGLO vector (Promega, Madison, WI, USA). VSMC and HUVEC were cultured for 12 h and transfected with various combinations of miR-21 mimics, NC mimics and pGL3-CASC7 3′-UTR WT or MUT. The dual-luciferase vector pmirGLO (Promega, Madison, WI, USA) was modified with the 3′-UTR of TLR4 and PI3K that contained a candidate miR-21 binding site or mutation thereof. Thereafter, pmirGLO PI3K and TLR4-WT or pmirGLO-PI3K and TLR4-Mut and miR-21 mimics or vector were co-transfected into HUVEC and VSMC. The Dual Luciferase Reporter Assay System was utilized for detecting relative luciferase activity.

### Western blotting (WB) assay

Total proteins were extracted from HUVEC and VSMC with RIPA lysis buffer. Thereafter, proteins (50 μg) were separated with 10% SDS-PAGE, followed by electrophoretic transfer to PVDF membranes (Beyotime, Shanghai, China). After blocking with 5% normal nonimmune goat serum, the membrane was exposed to primary antibodies against BCL-2 (Abcam, Cambridge, MA, USA), caspase-3 (Abcam, Cambridge, MA, USA), PI3K (Abcam, Cambridge, MA, USA), Akt (Abcam, Cambridge, MA, USA), TLR4 (Beyotime, Jiangsu, China), NF-κB (Abcam, Cambridge, MA, USA) or β-actin (Beyotime, Jiangsu, China) at 4°C overnight. After extensive washing, secondary antibodies (1:5000) were incubated with the membranes at ambient temperature for another 2 h. ECL luminous substrate was added to the membrane according to the manufacturer’s instructions, and protein bands were detected and quantified.

### Immunofluorescence assay

HUVEC and VSMC were fixed onto glass coverslips by treating with 4% paraformaldehyde for 10 min. Fixed cells treated with blocking solution at ambient temperature for 30 min, followed by incubation with primary antibody at 4°C overnight. Primary antibodies were against PI3K (Abcam, Cambridge, MA, USA), Akt (Abcam, Cambridge, MA, USA), TLR4 (Beyotime, Jiangsu, China), NF-κB (Abcam, Cambridge, MA, USA), or β-catenin (Beyotime, Jiangsu, China). Cells were washed and further cultured with an Alexa Fluor 488-labeled goat anti-rabbit IgG antibody (Boster Biotechnology, Wuhan, China) at ambient temperature for 1 h. Cell nuclei were counterstained with DAPI (Boster Biotechnology, Wuhan, China).

### Target gene prediction

Target miRNAs that might bind to CASC7 were predicted by analysis of the miRcode database (https://www.mircode.com/). The target genes of miR-21 were predicted with TargetScan software (http://www.targetscan.org/).

### Statistical analysis

GraphPad Prism 6.0 (GraphPad Software, Inc., CAPrism6, USA) and SPSS23.0 (SPSS, Inc., Chicago, IL, USA) were applied for performing statistical analyses. Data were expressed as mean ± SD. Means between different variables were compared by repeated-measures ANOVA. The receiver operator characteristic (ROC) curve was used to evaluate the utility of differential lncRNAs concentration in predicting AS. Differences were analyzed with Student's *t*-tests. ANOVA followed by a least significant difference test was conducted to compare among several groups. Differences with *P* < 0.05 were considered to be statistically significant.

### Availability of data and materials

The datasets used and analyzed during the current study are available from the corresponding author on reasonable request.

## RESULTS

### CASC7 is downregulated both in AS serum and in cellular models treated with ox-LDL

**Figure 1:**
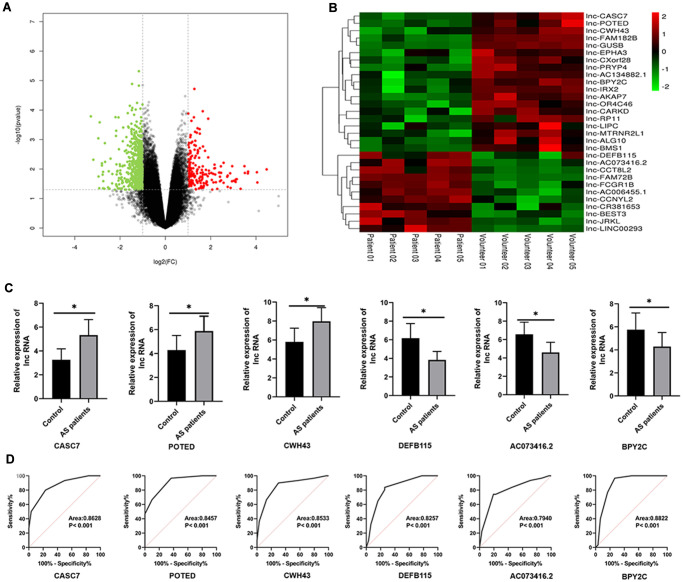
**Differential expression profile of lncRNAs between AS and healthy patients.** (**A**) The differential genes are represented by volcano plots. Gray points are genes with *P* values ≥ 0.05. Green point are genes with absolute fold changes ≥ 2 and *P* values < 0.05. The red dots are genes with fold changes ≥ 2 and *P* values < 0.05. (**B**) A heatmap showing differentially expressed lncRNAs. Green and red colors stand for down-regulation and up-regulation, respectively. (**C**) qRT-PCR was performed to detect the expression of lncRNAs in AS serum. (**D**) An ROC analysis of lncRNAs expression to differentiate between AS and healthy controls. Results are expressed as mean ± SD. ^*^P < 0.05.

The R language “limma” package was used for analyzing differentially expressed lncRNAs based on the screening criteria of |logFC| > 0 and *P* value < 0.05. As shown in a volcano plot (Figure 1A), a total of 1040 lncRNAs were significantly differentially expressed in AS serum as compared with volunteer serum, among which 458 were up-regulated and 582 were down-regulated. The 31 most highly differentially expressed lncRNAs were analyzed with a hierarchical clustering heat map based on screening criteria of |logFC| > 1 and *P* value < 0.01 (Figure 1B, Table 2).

**Table 2:** The top 31 differentially genes based on screening criteria of |logFC|>1 and *P* value < 0.01.

**ID**	***t***	**B**	**logFC**	**adj.*P*.Val**
lnc-DEFB115	−8.27	2.0457	−1.17	4.79E-06
lnc-AC073416.2	−7.44	1.5896	−1.47	1.3E-05
lnc-CCT8L2	−7.37	1.54285	−1	1.43E-05
lnc-FAM72B	−7.34	1.52507	−1.17	1.48E-05
lnc-FCGR1B	7.14	1.39872	−1.28	1.92E-05
lnc-AC006455.1	6.71	1.10959	−1.874	3.37E-05
lnc-CCNYL2	−6.36	0.8584	−1.73	5.39E-05
lnc-CR381653	−6.32	0.82902	−1.25	5.69E-05
lnc-BEST3	−6.23	0.75797	−1.01	6.47E-05
lnc-JRKL	−6.18	0.72019	−1.766	6.93E-05
lnc-LINC00293	−6.16	0.7041	−1.57	7.13E-05
lnc-CASC7	−6.04	0.61058	2.46	8.42E-05
lnc-POTED	−6.03	0.59556	2.381	8.65E-05
lnc-CWH43	−6.01	0.57874	2.1	8.91E-05
lnc-FAM182B	−6	0.5731	1.975	9E-05
lnc-GUSB	−5.95	0.53572	1.31	9.61E-05
lnc-EPHA3	−5.92	0.51151	1.765	0.0001
lnc-CXorf28	−5.92	0.50887	1.48	0.000101
lnc-PRYP4	−5.89	0.48334	1.8	0.000105
lnc-AC134882.1	5.87	0.46412	2.807	0.000109
lnc-BPY2C	5.86	0.46091	1.65	0.000109
lnc-IRX2	−5.82	0.42374	1.86	0.000117
lnc-AKAP7	5.78	0.38949	1.929	0.000124
lnc-OR4C46	−5.77	0.38039	1.904	0.000126
lnc-CARKD	−5.76	0.37491	1.66	0.000127
lnc-RP11	5.74	0.35655	1.01	0.000131
lnc-LIPC	5.73	0.34898	1.734	0.000133
lnc-MTRNR2L1	−5.72	0.3352	1.965	0.000136
lnc-ALG10	5.71	0.32676	1.91	0.000138
lnc-BMS1	5.68	0.30122	1.767	0.00944

We collected 30 serum samples from both normal volunteers and AS cases in order to further investigate the expression levels of the of the top three up-regulated and the top three down-regulated differential lncRNAs. According to the results of a qRT-PCR analysis, the levels of CASC7, POTED and CWH43 were significantly decreased in AS patients as compared with healthy volunteers (*P* < 0.05, Figure 1C). On the other hand, the levels of DEFB115, AC073416.2 and BPY2C here all significantly higher in AS patients as compared to healthy volunteers (*P* < 0.05, Figure 1C). A ROC analysis also showed that these lncRNAs exerted discriminatory capabilities that could be used to differentiate between patients with AS and healthy volunteers (*P* < 0.05; Figure 1D).

### CASC7 inhibits proliferation and promotes apoptosis of VSMC and suppresses the inflammatory response of HUVEC

**Figure 2:**
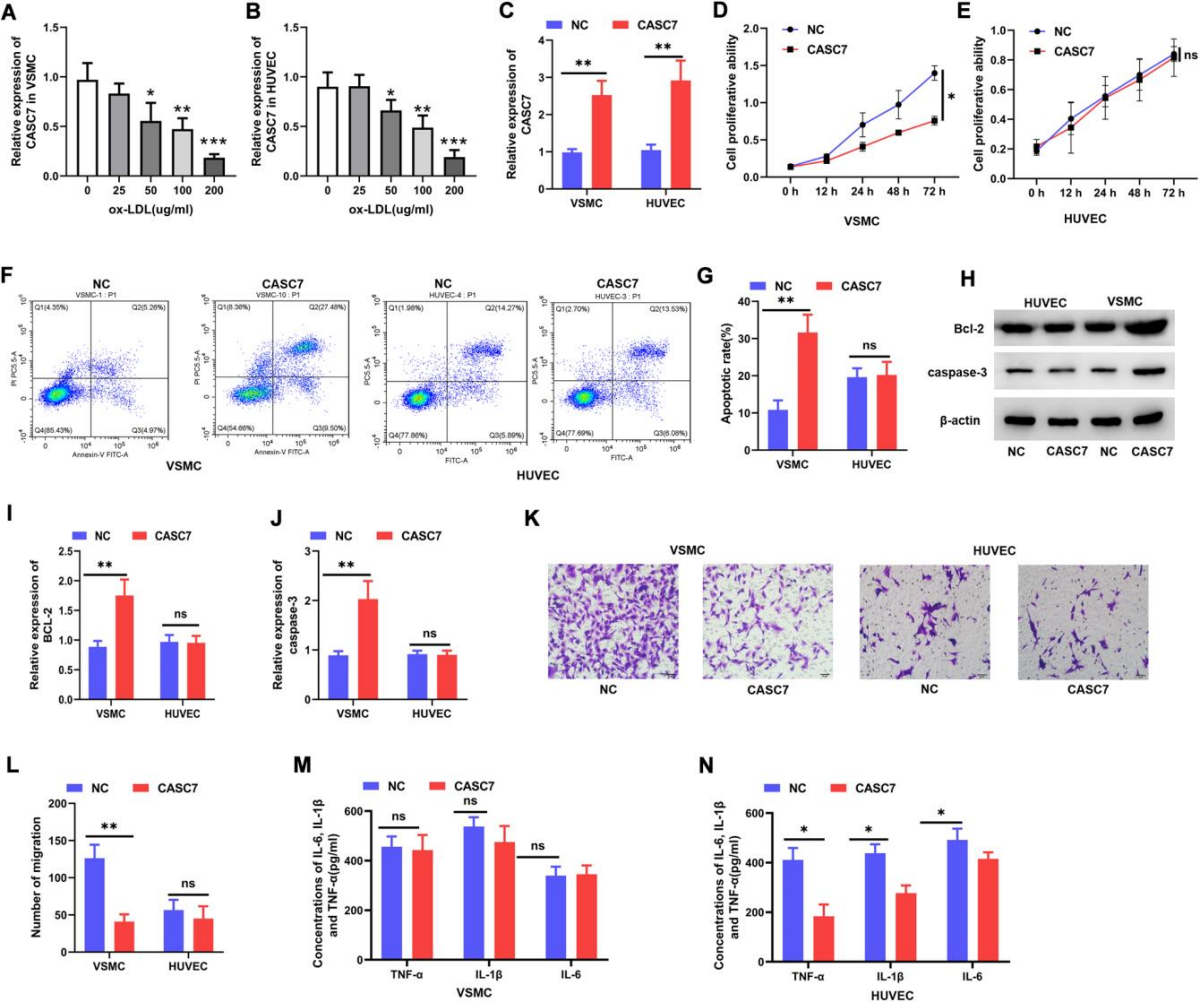
**CASC7 inhibits proliferation and promotes apoptosis of VSMC and suppresses inflammatory responses of HUVEC.** (**A**, **B**) qRT-PCR was carried out to measure CASC7 levels within ox-LDL-treated HUVEC and VSMC. (**C**) Expression of CASC7 in VSMC and HUVEC was determined with qRT-PCR. (**D**, **E**) Cell viability was evaluated by CCK-8 assays. (**F**, **G**). Flow cytometry was performed to analyze apoptosis in HUVEC and VSMC. (**H**–**J**) Caspase-3 and Bcl-2 protein expression was measured through Western blot assays. (**K**, **L**) Transwell assays were performed to evaluate cell invasion. (**M**, **N**) ELISA was conducted to detect expression of TNF-α, IL-6 and IL-1β. Typical images for HUVEC and VSMC (400×) are presented. Results are expressed as mean ± SD. ^*^P < 0.05, ^**^P < 0.01, ^***^P < 0.001.

Various doses of ox-LDL were administered to cultured HUVEC and VSMC, and CASC7 expression was quantified after 24 h. According to these *in vitro* analyses, the level of CASC7 declined markedly within VSMC and HUVEC following ox-LDL treatment in a dose-dependent manner (Figure 2A, 2B). The overexpression of CASC7 was induced via transfecting VSMC and HUVEC with pcDNA-CASC7. At 24 h post-transfection, the level of CASC7 was significantly increased in the CASC7 group in comparison with NC group, as determined by quantitative RT-PCR (*P* < 0.05, Figure 2C). In order to explore the effects of CASC7 on biological functions of cells treated with ox-LDL, CASC7-overexpressing cells were analyzed for proliferation, migration and apoptotic ability. According to the results of CCK-8, Transwell, and flow cytometric assays, upregulated CASC7 significantly decreased VSMC proliferation ability (*P* < 0.05, Figure 2D), significantly inhibited migration capacity (*P* < 0.05, Figure 2K, 2L), and significantly promoted apoptosis (*P* < 0.05, Figure 2F, 2G), respectively.

The protein levels of caspase-3 and Bcl-2 were elevated in CASC7-overexpressing VSMC relative to the NC group (*P* < 0.05, Figure 2H–2J). However, increasing CASC7 levels had no effect on cell proliferation (*P* < 0.05, Figure 2E), migration (*P* < 0.05, Figure 2K, 2L) or apoptosis (*P* < 0.05, Figure 2F, 2G) in HUVEC treated with ox-LDL. Similarly, after transfection with pcDNA-CASC7, there was no change in caspase-3 or Bcl-2 protein expression in ox-LDL-exposed HUVEC (*P* < 0.05, Figure 2H–2J). When exploring the effect of CASC7 on the inflammatory response of cells treated by ox-LDL, results demonstrated no influence of increased CASC7 on the levels of TNF-α, IL-1β or IL-6 in VSMC (*P* > 0.05, Figure 2M), but a significant decrease of these cytokines in HUVEC was found (*P* < 0.05, Figure 2N).

### CASC7 is a decoy of miR-21

**Figure 3:**
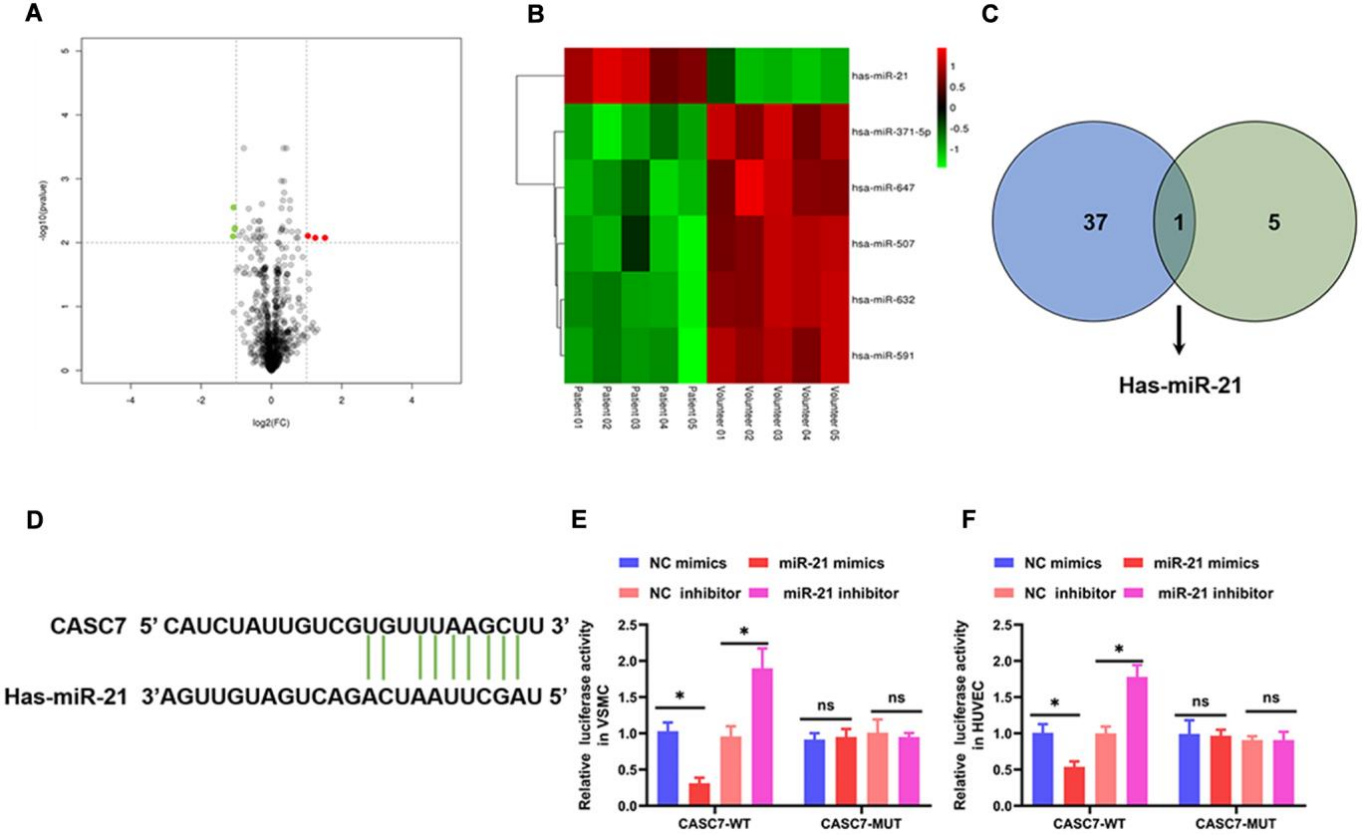
**CASC7 is a decoy of miR-21.** (**A**) The differential genes are represented by volcano plots. Gray points are genes with *P* values ≥ 0.01. Green point are genes with absolute fold changes ≥1 and *P* values < 0.01. The red dots are genes with fold changes ≥1 and *P* values < 0.01. (**B**) A heatmap showing differentially expressed miRNAs. Green and red colors stand for down-regulation and up-regulation, respectively. (**C**) Intersection analysis of target miRNAs of CASC7 and differentially expressed miRNAs. (**D**) The binding site of CASC7 in the 3′-UTR of miR-21 was predicted by TargetScan. (**E** and **F**) Luciferase activity of CASC7 as detected by a dual-luciferase reporter assay. Results are expressed as mean ± SD. ^*^P < 0.05.

To study the detailed mechanism by which CASC7 inhibited proliferation and promoted apoptosis of VSMC and suppressed the inflammatory response of HUVEC, the differentially expressed miRNAs were analyzed by the R package “limma” based on the screening criteria of |logFC| > 0 and *P* value < 0.01. These results identified six miRNAs that are differentially expressed in AS patients relative to health volunteers (Figure 3A, 3B, Table 3). Next, miRNAs that might bind to the CASC7 were predicted through miRcode. As listed in Supplementary Table 1, 37 miRNAs were found to potentially bind to CASC7. Subsequently, by analyzing the intersection of predicted CASC7-targeting miRNAs with the significantly differentially expressed miRNAs, we discovered that miR-21 was common to both sets (Figure 3C). To further determine whether miR-21 is the target of CASC7, a computer-based predictive database (LncBase V2.0) was used. This analysis also predicted miR-21 to be a candidate CASC7 target (Figure 3D).

**Table 3:** The 6 differentially genes based on screening criteria of |logFC|>0 and *P* value < 0.01.

**ID**	***t***	**B**	**logFC**	**adj.*P*.Val**
hsa-miR-632	10.495	5.7234	0.3457166	0.0003324
hsa-miR-371-5p	10.71069	5.8955	0.4273783	0.0003324
hsa-miR-21	−10.6326	5.8336	−0.7822816	0.0003324
hsa-miR-591	8.986339	4.4074	0.27949	0.0010807
hsa-miR-647	8.835394	4.264	0.356713	0.0010914
hsa-miR-507	8.192145	3.6262	0.3630077	0.0016456

In order to further validate the binding between CASC7 and miR-21, we constructed luciferase reporter genes under the control of a promoter containing either the WT 3′-UTR of CASC7 or a mutated version (MUT) of the 3′-UTR. In a luciferase reporter assay, mimics of miR-21 significantly reduced luciferase activity in VSMC (*P* < 0.05, Figure 3E) and HUVEC (*P* < 0.05, Figure 3F) in the CASC7-WT group. In addition, an miR-21 inhibitor increased the luciferase activity in VSMC (*P* < 0.05, Figure 3E) and HUVEC (*P* < 0.05, Figure 3F) in the CASC7-WT group, but not in the CASC7-MUT group (*P* > 0.05, Figure 3E, 3F).

### The miR-21 reverses the biological effects of CASC7 on VSMC and HUVEC

**Figure 4:**
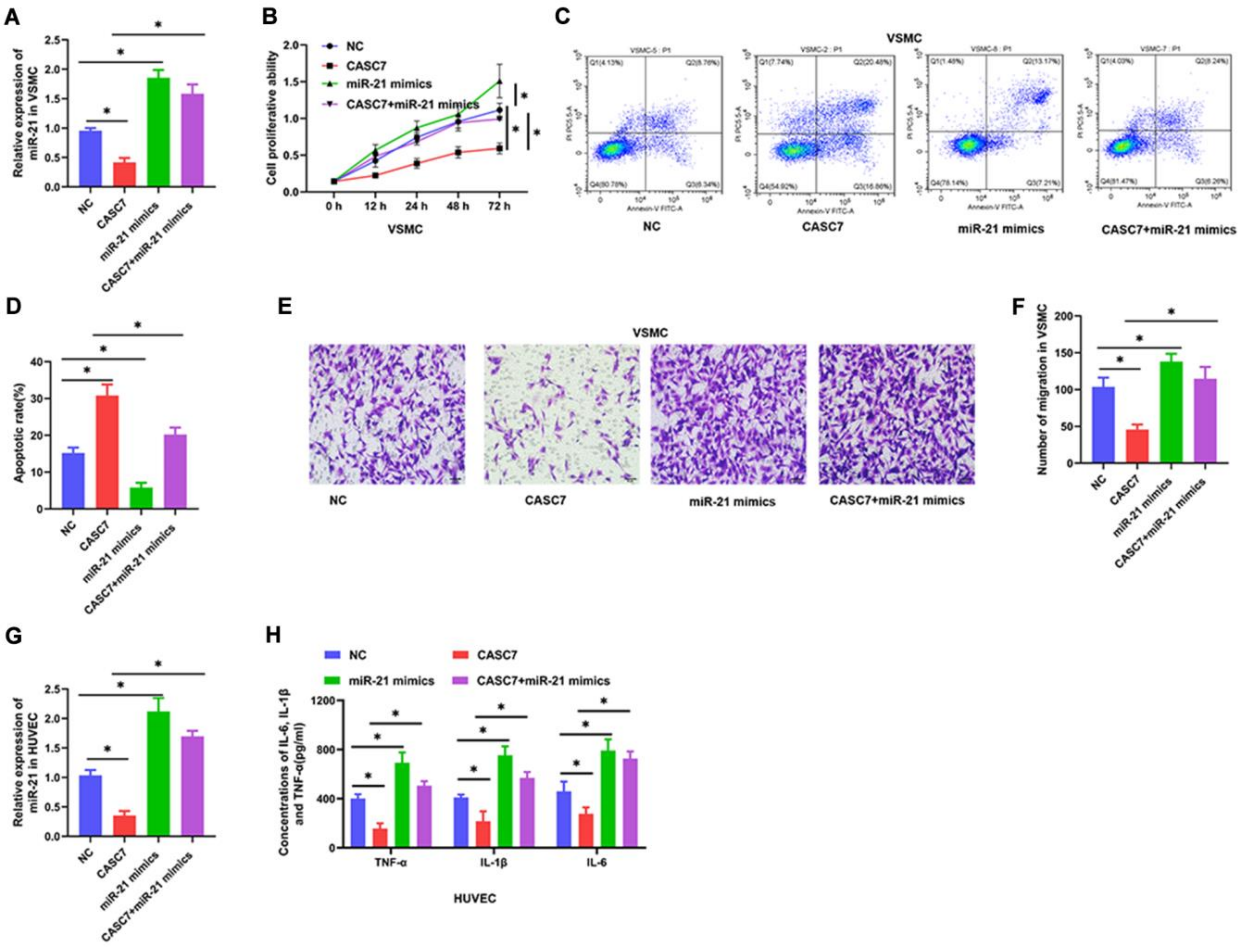
**miR-21 reverses the biological effects of CASC7 on VSMC and HUVEC.** (**A**) Expression of miR-21 in VSMC as detected by qRT-PCR. (**B**) Cell viability evaluated by CCK-8 assays. (**C** and **D**). Apoptosis rates of VSMC were tested by flow cytometry. (**E** and **F**) Invasion capability was evaluated by Transwell invasion assays. (**G**) Expression of miR-21 in HUVEC as detected by qRT-PCR. (**H**) Levels of inflammatory factors, including TNF-α, IL-1β and IL-6, were detected by ELISA. Photomicrographs show representative images of the VSMC and HUVEC (400×). Data are presented as mean ± SD. ^*^P < 0.05.

In order to confirm that CASC7 exerts its biological effects through miR-21, the concentration of miR-21 was increased by transfection with constructs that lead to the expression of miR-21 mimics. As shown in Figure 4A and 4G, CASC7 over-expression remarkably decreased expression of miR-21 (*P* < 0.05). Following transfection of the miR-21 mimic constructs, the level of miR-21 was increased in the miR-21 mimics group (*P* < 0.05, Figure 4A and 4G) when compared with the NC group. Moreover, we found that miR-21 over-expression completely blocked the effect of CASC7 in decreasing proliferation ability (*P* < 0.05, Figure 4B), inhibited migration ability (*P* < 0.05, Figure 4E, 4F) and promoted apoptosis (*P* < 0.05, Figure 4C, 4D) in VSMC. Finally, an exploration of the effect of miR-21 on the inflammatory response in HUVEC showed that increasing the expression of miR-21 led to significantly increased expression of TNF-α, IL-6 and IL-1β in HUVEC that were overexpressing both CASC7 and the miR-21 mimics as compared with HUVEC only overexpressing CASC7 (*P* < 0.05, Figure 4H).

### PI3K and TLR4 are the targets of different roles of miR-21

**Figure 5:**
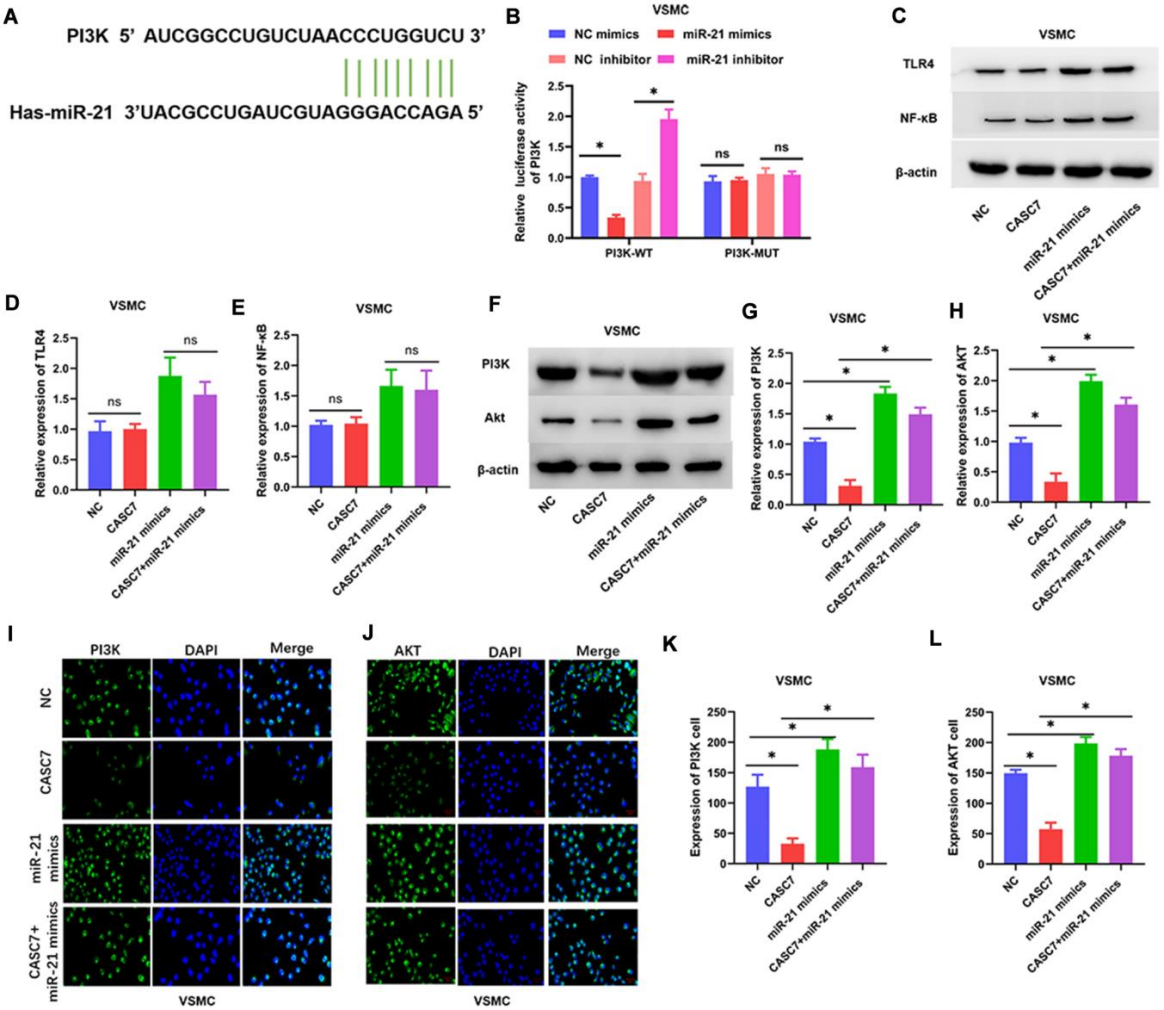
**The PI3K/Akt pathway is a target of miR-21.** (**A**) The binding site of miR-21 in the 3′-UTR of PI3K was predicted by TargetScan. (**B**) Luciferase activity of PI3K as detected by dual-luciferase reporter assay. (**C**–**H**) Expression of TLR4, NF-κB, PI3K and Akt examined by Western blot assays. (**I**–**L**) Luciferase activity of PI3K and Akt in VSMC examined by immunofluorescence assays. Representative images in VSMC (400×). Data are presented as mean ± SD. ^*^P < 0.05.

Several studies have shown that the PI3K/Akt and TLR4/NF-κB signal transduction pathways are important targets of miR-21 [29, 30]. Therefore, we further examined the interaction between miR-21 and the PI3K/Akt signaling pathway in VSMC. TargetScan 7.2 analysis suggested that miR-21 targeted PI3K (Figure 5A). However, in this study, the levels of expression of TLR4 and NF-κB were not found to be downregulated by CASC7 in VSMC treated with ox-LDL (*P* < 0.05, Figure 5C–5E). Therefore, the interaction of miR-21 with PI3K activity in VSMC was tested through luciferase reporter assays. These assays showed that the miR-21 mimics reduced translation of luciferase from an mRNA that contains a putative miR-21 target region from PI3K mRNA, whereas the miR-21 inhibitor increased the luciferase activity (*P* < 0.05, Figure 5B). Consistently, protein levels of PI3K and Akt were significantly reduced upon expression of CASC7, and this effect could be rescued by expression of miR-21 mimics in VSMC (*P* < 0.05, Figure 5F–5H). Additionally, Akt and PI3K levels were elevated in the miR-21 mimics group relative to the group in which both CASC7 and miR-21 were overexpressed, as revealed by an immunofluorescence assay in VSMC (*P* < 0.05, Figure 5I–5L).

**Figure 6:**
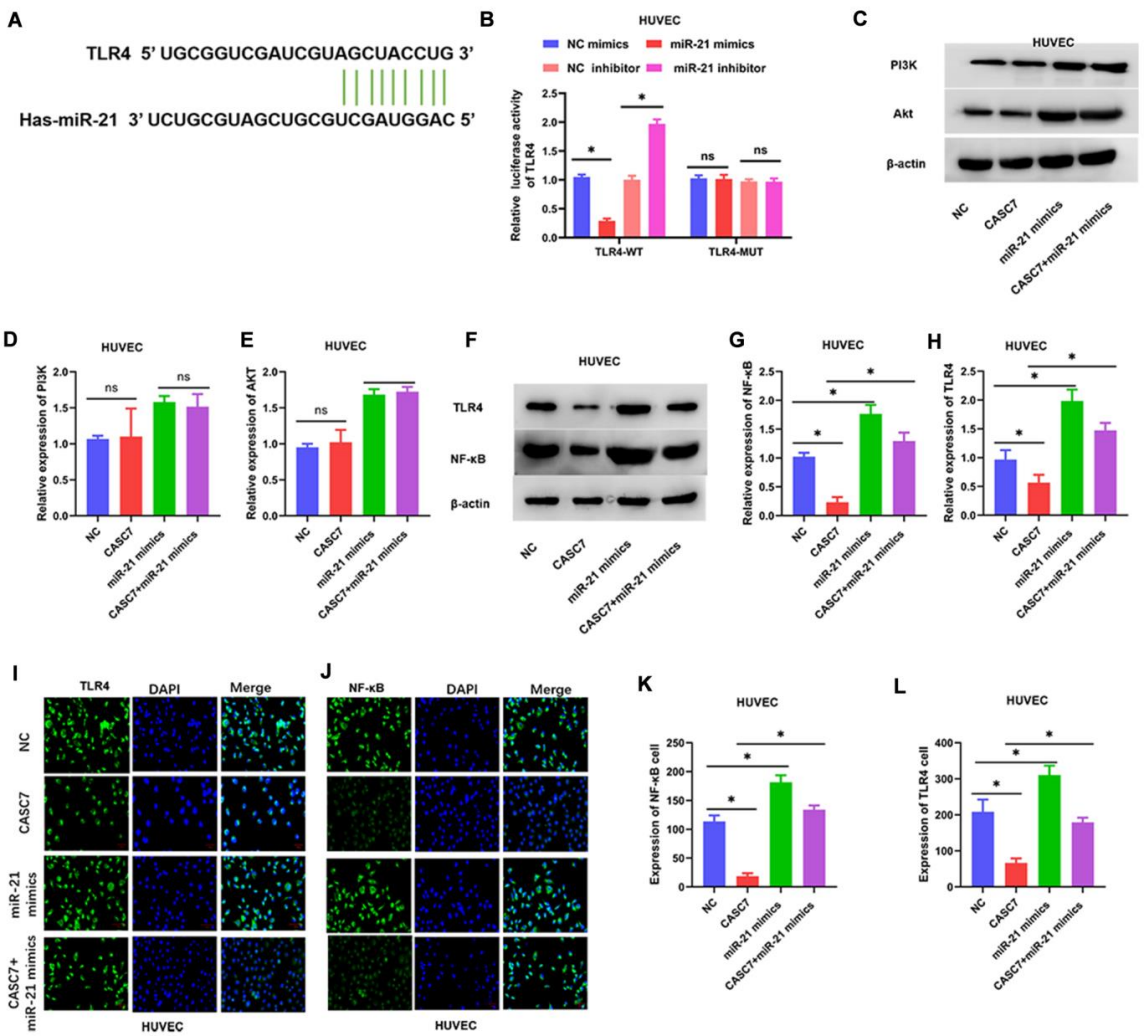
**The TLR4/NF-κB axis is a target of miR-21.** (**A**) The binding site of miR-21 in the 3′-UTR of TLR4 was predicted by TargetScan. (**B**) Luciferase activity of TLR4 as detected by dual-luciferase reporter assay. (**C**–**H**) Expression of TLR4, NF-κB, PI3K and Akt examined by Western blot assays. (**I**–**L**) Luciferase activity of TLR4 and NF-κB in HUVEC examined by immunofluorescence assays. Representative images of HUVEC (400×). Data are presented as mean ± SD. ^*^P < 0.05.

Levels of TLR4 and NF-κB, but not PI3K and Akt (*P* > 0.05, Figure 6C–6E), were decreased in HUVEC overexpressing CASC7 relative to the NC group. Similarly, another candidate miR-21 target predicted by the TargetScan software was TLR4 (Figure 6A). Accordingly, miR-21 mimics decreased TLR4-dependent luciferase activity within cells expressing wild-type TLR4, whereas an miR-21 inhibitor increased the luciferase activity downstream of TLR4 (*P* < 0.05, Figure 6B). According to Western blot assays, overexpression of CASC7 restrained the levels of TLR4 and NF-κB proteins, and this inhibitory effect of CASC7 was reversed by the expression of miR-21 mimics in HUVEC (*P* < 0.05, Figure 6F–6H). Similarly, NF-κB and TLR4 levels were elevated within cells expressing CASC7 and the miR-21 mimics relative to those expressing only CASC7, according to immunofluorescence assays (*P* < 0.05, Figure 6I–6L).

### Upregulation of PI3K and TLR4 blocks biological effects of CASC7

**Figure 7:**
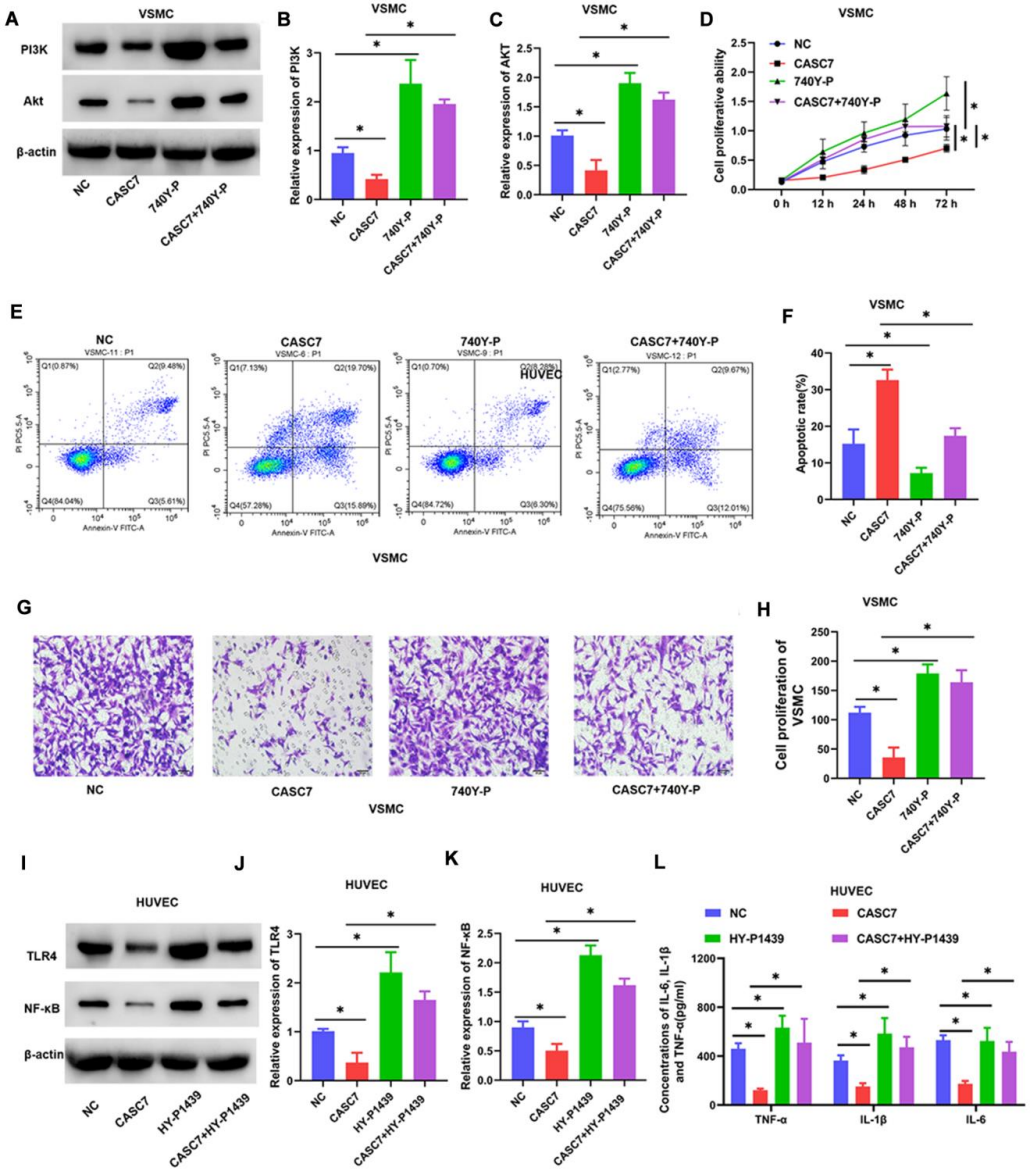
**Upregulated PI3K and TLR4 blocks the biological effects of CASC7 on VSMC and HUVEC, respectively.** (**A**–**C**) Expression levels of PI3K and Akt in VSMC were determined by Western blot assays. (**D**) Cell viability of VSMC cells was evaluated by CCK-8 assays. (**E**, **F**) Apoptosis rates of VSMC were tested by flow cytometry. (**G**, **H**) Invasion capability was evaluated by Transwell invasion assays. (**I**–**K**) Expression of TLR4 and NF-κB in HUVEC were quantified by Western blot assays. (**L**) Levels of inflammatory factors, including TNF-α, IL-1β and IL-6, were detected by ELISA. Photomicrographs show representative images of the VSMC and HUVEC (400×). Data are presented as mean ± SD. ^*^P < 0.05.

To investigate the role of PI3K in the regulation of VSMC by CASC7, the specific PI3K agonist 740Y-P was used to activate the PI3K/Akt signaling pathway. As shown in Figure 6, 740Y-P increased the protein levels of PI3K and Akt (*P* < 0.05, Figure 7A–7C). In addition, treatment with 740Y-P significantly inhibited the effect of expression of CASC7 on decreasing the proliferation (*P* < 0.05, Figure 7D) and the migration (*P* < 0.05, Figure 7G, 7H) of VSMC, and the promotion of apoptosis was also reversed by the PI3K agonist in VSMC (*P* < 0.05, Figure 7E, 7F).

Similarly, when we explored CASC7-mediated suppression of the inflammatory response of HUVEC by the TLR4/NF-κB signaling pathway, we found that the TLR4 agonist HY-P1439 increased the levels of TLR4 and NF-κB proteins (*P* < 0.05, Figure 7I–7K). Overexpression of TLR4 also increased expression of TNF-α, IL-6 and IL-1β within HUVEC relative to HUVEC that express CASC7 and are treated with HY-P1439 (*P* < 0.05, Figure 7L).

## DISCUSSION

AS represents a complex pathophysiological process that is a frequent cause of cardiovascular diseases. Previous research has demonstrated that the aberrant migration, proliferation, and apoptosis of VSMC and the inflammatory response of endothelial cells are associated with progression of early AS [3]. LncRNAs have been proven to perform vital roles in the genesis, prevention and management of AS [31, 32]. The present work identifies a total of 1040 differentially-expressed lncRNAs that characterize the development of AS. One particular lncRNA, CASC7, is downregulated in the serum of AS patients. In studies *in vitro*, we adopted ox-LDL-induced HUVEC and VSMC as a model of AS, and we found that the CASC7/miR-21 axis presents different biological effects and mechanisms of action in these two models. Briefly, CASC7 inhibited proliferation and promoted apoptosis of VSMC by regulating the miR-21/PI3K-AKT axis, but increased CASC7 expression suppressed the inflammatory response of HUVEC by sponging miR-21 to inhibit the TLR4/NF-κB signaling pathway.

CASC7 is known to participate in several other disorders, including colon cancer [21], asthma [18], glioma [16] and myocardial ischemia–reperfusion injury [19]. However, it was unclear whether CASC7 was involved in the pathological process of AS. In the present work, we found that CASC7 is lower in the serum of AS patients and that the level of expression could be used to differentiate between AS patients and healthy volunteers. The findings suggest that CASC7 might play an important role in the progression of AS.

To build a cellular model of AS, ox-LDL was presented to VSMC and HUVEC. CASC7 expression was obviously decreased in VSMC and HUVEC after stimulation with ox-LDL. Upregulated CASC7 significantly decreased the proliferation ability and migration capacity, and promoted apoptosis, in the model VSMC. Meanwhile, upregulated CASC7 suppressed the levels of inflammatory cytokines, including TNF-α, IL-1β and IL-6, in HUVEC treated with ox-LDL. We found that CASC7 inhibited proliferation and migration, which is consistent with the research of Zhang et al., who also reported that CASC7 inhibits the proliferation and migration of colon cancer cells [21]. However, this inhibitory effect was not observed in HUVEC treated with ox-LDL. This suggests that the inhibition of proliferation and invasion by CASC7 is cell-type specific.

Others have reported that CASC7 functions as an miR-21 decoy in colon cancer and myocardial ischemia-reperfusion [19, 21]. Here, we also found that miR-21 was a potential target of CASC7. A luciferase reporter assay demonstrated that miR-21 performed in direct opposition to CASC7. Interestingly, miR-21 expression was found to be decreased when transfected with CASC7 both in VSMC and HUVEC treated with ox-LDL. Further functional experiments suggested that overexpression of miR-21 completely blocked the impact of CASC7 on the decreasing of the proliferation ability, the inhibition of migration capacity, and the promotion of apoptosis in VSMC. Similarly, upregulated miR-21 significantly increased levels of TNF-α, IL-1β and IL-6 in HUVEC after transfection with CASC7. This suggests that CASC7 inhibited proliferation and promoted apoptosis of VSMC, and suppressed the inflammatory response of HUVEC by inhibiting miR-21.

In the PI3K/Akt signaling pathway, PI3K is activated by a G-protein coupled receptor or protein tyrosine kinase receptor, and then phosphatidylinositol-3-phosphate (PIP3), a second messenger, is produced in the cell membrane. PIP3 further binds to the signaling protein molecule AKT through PH domains, activating AKT by inducing it to migrate from the cytoplasm to the cell membrane [33]. Numerous studies have shown that this pathway modulates cell proliferation, differentiation, apoptosis, motility, and glucose metabolism [34–36]. In the current work, we discovered that overexpression of miR-21 could increase the levels of both PI3K and Akt. Meanwhile, the PI3K/Akt signaling pathway is the target of CASC7/miR-21 axis in VSMC, and the inhibitory effect of CASC7 in decreasing proliferation ability, inhibited migration capacity, and promoted apoptosis was reversed by PI3K agonist in VSMC. These results suggest that CASC7 inhibits proliferation and promotes apoptosis of VSMC by regulating the miR-21/PI3K-AKT axis.

Activated Akt, as a key molecule in the PI3K/Akt signaling pathway, is known to cause a series of downstream phosphorylation cascade reactions and to induce interactions between target proteins, including mammalian target of rapamycin, proteins of the Bcl-2 family, glycogen synthase kinase 3, caspase-9 and fork head-related transcription factor [37], which are involved in various processes, including the cell cycle, apoptosis, migration, proliferation and angiogenesis [35, 38, 39]. The regulation of the miR-21/PI3K-AKT axis by CASC7, then, provides additional avenues for investigation of the pathological processes underlying AS.

The TLR4/NF-κB signal pathway has been reported to be involved in inflammatory responses in central nervous system disease, ulcerative colitis and cancer [40–42]. In this study, we found that CASC7 correlates with decreased levels of TLR4 and NF-κB and that the TLR4/NF-κB signaling pathway is an important target of the CASC7/miR-21 axis in HUVEC. TLR4 blocks the suppressive effects of CASC7 on the inflammatory response in HUVEC. Mechanistically, activated TLR4 can bind to the TLR domain at the carboxyl terminus of MyD88 and activate NF-κB kinase through a series of phosphorylation reactions. NF-κB kinase can inhibit the ubiquitination and degradation of NF-κB, thereby activating NF-κB [43, 44]. Activated NF-κB enters the nucleus from the cytoplasm to activate the transcription and translation of cytokines, and finally promote the secretion of TNF-α, IL6 and IL-β [45–47].

In summary, we showed that CASC7 was downregulated in AS patients. Furthermore, CASC7 inhibited proliferation and promoted apoptosis of VSMC by regulating the miR-21/PI3K-AKT axis, and upregulated CASC7 suppressed the inflammatory response of HUVEC by sponging miR-21 to inhibit the TLR4/NF-κB signaling pathway. These results might provide a potential new target for the diagnosis and treatment of AS.

## Supplementary Materials

Supplementary Table 1

## References

[1] Collins AR, Lyon CJ, Xia X, Liu JZ, Tangirala RK, Yin F, Boyadjian R, Bikineyeva A, Praticò D, Harrison DG, Hsueh WA. Age-accelerated atherosclerosis correlates with failure to upregulate antioxidant genes. Circ Res. 2009; 104:e42–54. 10.1161/CIRCRESAHA.108.188771 19265038

[2] Wolf D, Ley K. Immunity and Inflammation in Atherosclerosis. Circ Res. 2019; 124:315–27. 10.1161/CIRCRESAHA.118.313591 30653442 PMC6342482

[3] Bennett MR, Sinha S, Owens GK. Vascular Smooth Muscle Cells in Atherosclerosis. Circ Res. 2016; 118:692–702. 10.1161/CIRCRESAHA.115.306361 26892967 PMC4762053

[4] Gimbrone MA Jr, García-Cardeña G. Endothelial Cell Dysfunction and the Pathobiology of Atherosclerosis. Circ Res. 2016; 118:620–36. 10.1161/CIRCRESAHA.115.306301 26892962 PMC4762052

[5] Dimmeler S, Hermann C, Zeiher AM. Apoptosis of endothelial cells. Contribution to the pathophysiology of atherosclerosis? Eur Cytokine Netw. 1998; 9:697–8. 9889419

[6] Gao S, Zhao D, Wang M, Zhao F, Han X, Qi Y, Liu J. Association Between Circulating Oxidized LDL and Atherosclerotic Cardiovascular Disease: A Meta-analysis of Observational Studies. Can J Cardiol. 2017; 33:1624–32. 10.1016/j.cjca.2017.07.015 29173602

[7] Suciu CF, Prete M, Ruscitti P, Favoino E, Giacomelli R, Perosa F. Oxidized low density lipoproteins: The bridge between atherosclerosis and autoimmunity. Possible implications in accelerated atherosclerosis and for immune intervention in autoimmune rheumatic disorders. Autoimmun Rev. 2018; 17:366–75. 10.1016/j.autrev.2017.11.028 29425936

[8] Kattoor AJ, Kanuri SH, Mehta JL. Role of Ox-LDL and LOX-1 in Atherogenesis. Curr Med Chem. 2019; 26:1693–700. 10.2174/0929867325666180508100950 29737246

[9] Bian W, Jing X, Yang Z, Shi Z, Chen R, Xu A, Wang N, Jiang J, Yang C, Zhang D, Li L, Wang H, Wang J, et al. Downregulation of LncRNA NORAD promotes Ox-LDL-induced vascular endothelial cell injury and atherosclerosis. Aging (Albany NY). 2020; 12:6385–400. 10.18632/aging.103034 32267831 PMC7185106

[10] Song Y, Hou M, Li Z, Luo C, Ou JS, Yu H, Yan J, Lu L. TLR4/NF-κB/Ceramide signaling contributes to Ox-LDL-induced calcification of human vascular smooth muscle cells. Eur J Pharmacol. 2017; 794:45–51. 10.1016/j.ejphar.2016.11.029 27876618

[11] Tao K, Hu Z, Zhang Y, Jiang D, Cheng H. LncRNA CASC11 improves atherosclerosis by downregulating IL-9 and regulating vascular smooth muscle cell apoptosis and proliferation. Biosci Biotechnol Biochem. 2019; 83:1284–8. 10.1080/09168451.2019.1597621 30915898

[12] He X, Lian Z, Yang Y, Wang Z, Fu X, Liu Y, Li M, Tian J, Yu T, Xin H. Long Non-coding RNA PEBP1P2 Suppresses Proliferative VSMCs Phenotypic Switching and Proliferation in Atherosclerosis. Mol Ther Nucleic Acids. 2020; 22:84–98. 10.1016/j.omtn.2020.08.013 32916601 PMC7490454

[13] Cui C, Wang X, Shang XM, Li L, Ma Y, Zhao GY, Song YX, Geng XB, Zhao BQ, Tian MR, Wang HL. lncRNA 430945 promotes the proliferation and migration of vascular smooth muscle cells via the ROR2/RhoA signaling pathway in atherosclerosis. Mol Med Rep. 2019; 19:4663–72. 10.3892/mmr.2019.10137 30957191 PMC6522828

[14] Wang M, Li C, Zhang Y, Zhou X, Liu Y, Lu C. LncRNA MEG3-derived miR-361-5p regulate vascular smooth muscle cells proliferation and apoptosis by targeting ABCA1. Am J Transl Res. 2019; 11:3600–609. 31312370 PMC6614649

[15] Shan H, Guo D, Zhang S, Qi H, Liu S, Du Y, He Y, Wang B, Xu M, Yu X. SNHG6 modulates oxidized low-density lipoprotein-induced endothelial cells injury through miR-135a-5p/ROCK in atherosclerosis. Cell Biosci. 2020; 10:4. 10.1186/s13578-019-0371-2 31921409 PMC6947907

[16] Gong X, Liao X, Huang M. LncRNA CASC7 inhibits the progression of glioma via regulating Wnt/β-catenin signaling pathway. Pathol Res Pract. 2019; 215:564–70. 10.1016/j.prp.2019.01.018 30661904

[17] Zhou X, Lu H, Li F, Han L, Zhang H, Jiang Z, Dong Q, Chen X. LncRNA cancer susceptibility candidate (CASC7) upregulates phosphatase and tensin homolog by downregulating miR-10a to inhibit neuroblastoma cell proliferation. Neuroreport. 2020; 31:381–6. 10.1097/WNR.0000000000001411 32101951

[18] Liu JH, Li C, Zhang CH, Zhang ZH. LncRNA-CASC7 enhances corticosteroid sensitivity via inhibiting the PI3K/AKT signaling pathway by targeting miR-21 in severe asthma. Pulmonology. 2020; 26:18–26. 10.1016/j.pulmoe.2019.07.001 31412983

[19] Liao B, Gao F, Lin F, Yang S, Xu Z, Dong S. LncRNA CASC7 inhibits myocardial apoptosis in myocardial ischemia-reperfusion rats by regulating MiR-21 expression. Panminerva Med. 2019. [Epub ahead of print]. 10.23736/S0031-0808.19.03728-5 31362482

[20] Bai Y, Zhang Q, Su Y, Pu Z, Li K. Modulation of the Proliferation/Apoptosis Balance of Vascular Smooth Muscle Cells in Atherosclerosis by lncRNA-MEG3 via Regulation of miR-26a/Smad1 Axis. Int Heart J. 2019; 60:444–50. 10.1536/ihj.18-195 30745534

[21] Zhang Z, Fu C, Xu Q, Wei X. Long non-coding RNA CASC7 inhibits the proliferation and migration of colon cancer cells via inhibiting microRNA-21. Biomed Pharmacother. 2017; 95:1644–53. 10.1016/j.biopha.2017.09.052 28954383

[22] Canfrán-Duque A, Rotllan N, Zhang X, Fernández-Fuertes M, Ramírez-Hidalgo C, Araldi E, Daimiel L, Busto R, Fernández-Hernando C, Suárez Y. Macrophage deficiency of miR-21 promotes apoptosis, plaque necrosis, and vascular inflammation during atherogenesis. EMBO Mol Med. 2017; 9:1244–62. 10.15252/emmm.201607492 28674080 PMC5582411

[23] Zhu J, Liu B, Wang Z, Wang D, Ni H, Zhang L, Wang Y. Exosomes from nicotine-stimulated macrophages accelerate atherosclerosis through miR-21-3p/PTEN-mediated VSMC migration and proliferation. Theranostics. 2019; 9:6901–19. 10.7150/thno.37357 31660076 PMC6815950

[24] Lu JM, Zhang ZZ, Ma X, Fang SF, Qin XH. Repression of microRNA-21 inhibits retinal vascular endothelial cell growth and angiogenesis via PTEN dependent-PI3K/Akt/VEGF signaling pathway in diabetic retinopathy. Exp Eye Res. 2020; 190:107886. 10.1016/j.exer.2019.107886 31759996

[25] Zhao J, Liu B, Li C. Knockdown of long noncoding RNA GAS5 protects human cardiomyocyte-like AC16 cells against high glucose-induced inflammation by inhibiting miR-21-5p-mediated TLR4/NF-κB signaling. Naunyn Schmiedebergs Arch Pharmacol. 2020; 393:1541–7. 10.1007/s00210-019-01795-z 31865425

[26] Wang N, Zhang X, Ma Z, Niu J, Ma S, Wenjie W, Chen J. Combination of tanshinone IIA and astragaloside IV attenuate atherosclerotic plaque vulnerability in ApoE(-/-) mice by activating PI3K/AKT signaling and suppressing TRL4/NF-κB signaling. Biomed Pharmacother. 2020; 123:109729. 10.1016/j.biopha.2019.109729 31887543

[27] Qi JC, Liu PG, Wang C, Zheng AD, Wan Z. Tacrolimus protects vascular endothelial cells from injuries caused by Ox-LDL by regulating endoplasmic reticulum stress. Eur Rev Med Pharmacol Sci. 2017; 21:3966–73. 28975964

[28] Liu J, Yao S, Wang S, Jiao P, Song G, Yu Y, Zhu P, Qin S. D-4F, an apolipoprotein A-I mimetic peptide, protects human umbilical vein endothelial cells from oxidized low-density lipoprotein-induced injury by preventing the downregulation of pigment epithelium-derived factor expression. J Cardiovasc Pharmacol. 2014; 63:553–61. 10.1097/FJC.0000000000000080 24709637

[29] Lin F, Yin HB, Li XY, Zhu GM, He WY, Gou X. Bladder cancer cell-secreted exosomal miR-21 activates the PI3K/AKT pathway in macrophages to promote cancer progression. Int J Oncol. 2020; 56:151–64. 10.3892/ijo.2019.4933 31814034 PMC6910194

[30] Pan YQ, Li J, Li XW, Li YC, Li J, Lin JF. Effect of miR-21/TLR4/NF-κB pathway on myocardial apoptosis in rats with myocardial ischemia-reperfusion. Eur Rev Med Pharmacol Sci. 2018; 22:7928–37. 10.26355/eurrev_201811_16420 30536340

[31] Pan JX. LncRNA H19 promotes atherosclerosis by regulating MAPK and NF-kB signaling pathway. Eur Rev Med Pharmacol Sci. 2017; 21:322–8. 28165553

[32] Hu YW, Guo FX, Xu YJ, Li P, Lu ZF, McVey DG, Zheng L, Wang Q, Ye JH, Kang CM, Wu SG, Zhao JJ, Ma X, et al. Long noncoding RNA NEXN-AS1 mitigates atherosclerosis by regulating the actin-binding protein NEXN. J Clin Invest. 2019; 129:1115–28. 10.1172/JCI98230 30589415 PMC6391138

[33] Ersahin T, Tuncbag N, Cetin-Atalay R. The PI3K/AKT/mTOR interactive pathway. Mol Biosyst. 2015; 11:1946–54. 10.1039/c5mb00101c 25924008

[34] Gong C, Ai J, Fan Y, Gao J, Liu W, Feng Q, Liao W, Wu L. NCAPG Promotes The Proliferation Of Hepatocellular Carcinoma Through PI3K/AKT Signaling. Onco Targets Ther. 2019; 12:8537–52. 10.2147/OTT.S217916 31802891 PMC6801502

[35] Feng FB, Qiu HY. Effects of Artesunate on chondrocyte proliferation, apoptosis and autophagy through the PI3K/AKT/mTOR signaling pathway in rat models with rheumatoid arthritis. Biomed Pharmacother. 2018; 102:1209–20. 10.1016/j.biopha.2018.03.142 29710540

[36] Schultze SM, Hemmings BA, Niessen M, Tschopp O. PI3K/AKT, MAPK and AMPK signalling: protein kinases in glucose homeostasis. Expert Rev Mol Med. 2012; 14:e1. 10.1017/S1462399411002109 22233681

[37] Yudushkin I. Getting the Akt Together: Guiding Intracellular Akt Activity by PI3K. Biomolecules. 2019; 9:67. 10.3390/biom9020067 30781447 PMC6406913

[38] Chai C, Song LJ, Han SY, Li XQ, Li M. MicroRNA-21 promotes glioma cell proliferation and inhibits senescence and apoptosis by targeting SPRY1 via the PTEN/PI3K/AKT signaling pathway. CNS Neurosci Ther. 2018; 24:369–80. 10.1111/cns.12785 29316313 PMC6489721

[39] Karar J, Maity A. PI3K/AKT/mTOR Pathway in Angiogenesis. Front Mol Neurosci. 2011; 4:51. 10.3389/fnmol.2011.00051 22144946 PMC3228996

[40] Zusso M, Lunardi V, Franceschini D, Pagetta A, Lo R, Stifani S, Frigo AC, Giusti P, Moro S. Ciprofloxacin and levofloxacin attenuate microglia inflammatory response via TLR4/NF-kB pathway. J Neuroinflammation. 2019; 16:148. 10.1186/s12974-019-1538-9 31319868 PMC6637517

[41] Wang G, Xu B, Shi F, Du M, Li Y, Yu T, Chen L. Protective Effect of Methane-Rich Saline on Acetic Acid-Induced Ulcerative Colitis via Blocking the TLR4/NF-*κ*B/MAPK Pathway and Promoting IL-10/JAK1/STAT3-Mediated Anti-inflammatory Response. Oxid Med Cell Longev. 2019; 2019:7850324. 10.1155/2019/7850324 31182999 PMC6512011

[42] Yao D, Dong M, Dai C, Wu S. Inflammation and Inflammatory Cytokine Contribute to the Initiation and Development of Ulcerative Colitis and Its Associated Cancer. Inflamm Bowel Dis. 2019; 25:1595–602. 10.1093/ibd/izz149 31287863

[43] Su Q, Li L, Sun Y, Yang H, Ye Z, Zhao J. Effects of the TLR4/Myd88/NF-κB Signaling Pathway on NLRP3 Inflammasome in Coronary Microembolization-Induced Myocardial Injury. Cell Physiol Biochem. 2018; 47:1497–508. 10.1159/000490866 29940584

[44] Yue Y, Liu X, Li Y, Xia B, Yu W. The role of TLR4/MyD88/NF-κB pathway in periodontitis-induced liver inflammation of rats. Oral Dis. 2021; 27:1012–21. 10.1111/odi.13616 32853444 PMC8247295

[45] Gehrke N, Hövelmeyer N, Waisman A, Straub BK, Weinmann-Menke J, Wörns MA, Galle PR, Schattenberg JM. Hepatocyte-specific deletion of IL1-RI attenuates liver injury by blocking IL-1 driven autoinflammation. J Hepatol. 2018; 68:986–95. 10.1016/j.jhep.2018.01.008 29366909

[46] Cowland JB, Sørensen OE, Sehested M, Borregaard N. Neutrophil gelatinase-associated lipocalin is up-regulated in human epithelial cells by IL-1 beta, but not by TNF-alpha. J Immunol. 2003; 171:6630–9. 10.4049/jimmunol.171.12.6630 14662866

[47] Nyati KK, Masuda K, Zaman MM, Dubey PK, Millrine D, Chalise JP, Higa M, Li S, Standley DM, Saito K, Hanieh H, Kishimoto T. TLR4-induced NF-κB and MAPK signaling regulate the IL-6 mRNA stabilizing protein Arid5a. Nucleic Acids Res. 2017; 45:2687–703. 10.1093/nar/gkx064 28168301 PMC5389518

