# Supplementary material for: lncRNA CASC7 regulates pathological progression of ox-LDL-stimulated atherosclerotic cell models via sponging miR-21 and regulating PI3K/Akt and TLR4/NF-κB signaling pathways
Source: Aging (Albany NY). 2021 Dec 9;13(23):25408–25. doi: 10.18632/aging.203757 (PMC8714147; doi:10.18632/aging.203757)
Supplement: Supplementary Table 1 [file aging-13-203757-s001.pdf]

SUPPLEMENTARY TABLE

Supplementary Table 1. The potential target miRNAs of CASC7.

Family	Seed position	Seed type	Transcript region	Repeat	Conservation		
miR-7/7ab	chr21:46411004	7-mer-m8	ncRNA	no	67%	4%	0%
miR-138/138ab	chr21:46410327	7-mer-A1	ncRNA	no	11%	0%	0%
miR-143/1721/4770	chr21:46409908	7-mer-m8	ncRNA	no	67%	0%	0%
miR-181abcd/4262	chr21:46409833	7-mer-m8	ncRNA	yes	67%	0%	0%
miR-183	chr21:46409996	7-mer-m8	ncRNA	no	44%	0%	0%
miR-18ab/4735-3p	chr21:46410316	8-mer	ncRNA	no	33%	0%	0%
miR-193/193b/193a-3p	chr21:46410634	7-mer-m8	ncRNA	no	0%	0%	0%
miR-194	chr21:46410268	7-mer-A1	ncRNA	yes	11%	0%	0%
miR-1ab/206/613	chr21:46410043	7-mer-m8	ncRNA	no	44%	0%	0%
miR-203	chr21:46409786	7-mer-m8	ncRNA	yes	22%	0%	0%
miR-21	chr21:46409898	7-mer-A1	ncRNA	no	11%	0%	0%
miR-214/761/3619-5p	chr21:46411708	7-mer-m8	ncRNA	no	0%	0%	0%
miR-122/122a/1352	chr21:46411197	7-mer-m8	ncRNA	no	44%	0%	0%
miR-27abc/27a-3p	chr21:46410493	7-mer-m8	ncRNA	no	22%	0%	0%
miR-103a/107/107ab	chr21:46410250	7-mer-A1	ncRNA	yes	44%	0%	0%
miR-124/124ab/506	chr21:46410230	7-mer-m8	ncRNA	yes	56%	0%	0%
miR-128/128ab	chr21:46410494	8-mer	ncRNA	no	22%	0%	0%
